# Our Unfortunate Insane

**Published:** 1909-04

**Authors:** G. I. King

**Affiliations:** Elgin, Texas


### TEXAS MEDICAL JOURNAL.

Established July, 1885.

F. E. DANIEL, M. D., - - - - - Editor, Publisher and Proprietor

PUBLISHED MONTHLY.—SUBSCRIPTION $1.00 A YEAR.

VOL. XXIV. AUSTIN, APRIL, 1909. No. 10.

The publisher is not responsible for the views of contributors.

### Original Articles.

For Texas Medical Journal.

### Our Unfortunate Insane.

BY DR. G. I. KING, ELGIN, TEXAS.

The method of procedure and the treatment of the unfortunate insane of Texas, after conviction, has recently been the subject of much unfavorable criticism. Whether that criticism is just or unjust is a matter we propose to discuss, leaving you to judge for yourselves when the facts are presented.

THE ADJUDICATION.

The first thing necessary to do in order to start the machinery of the law in motion in regards to an insane person or lunatic is for some one interested to go before the county judge or a justice of the peace and make information in writing and under oath that such person is a lunatic or *non compos mentis* and that the welfare of himself or others requires that he be placed under restraint. When this information under oath is filed, then the county judge designates a place and date for a hearing of the complaint and a warrant is issued directing the sheriff or any constable of the county to have before the court on such date and at such place the person against whom the information has been filed. This being done, a jury of six competent jurors of the county are subpœnaed to appear at the place and on the date named, for the hearing to try the accused and to judge whether or not he or she is a lunatic or *non compos mentis.* The jury are required to take the following oath: "You and each of you do solemnly swear that upon all of the issues abbout to be submitted to you in the matter of the State of Texas against.........................................................., you will a true verdict render according to the evidence, so help you God." After taking this oath and hearing the testimony the following issues are submitted to the jury by the court:

1. Is the defendant of unsound mind?
2. If the defendant is of unsound mind, is it necessary that he should be placed under restraint?
3. Give the age and nativity of the defendant.
4. How many attacks of insanity has he had, and how long has the present attack existed?
5. Is insanity hereditary in the family of the defendant, or not?
6. What estate has the defendant?
7. If no estate, who is legally liable for his support?

If they find the first and second special issues as above submitted, in the affirmative, it is the duty of the court to render judgment, adjudging the defendant to be a lunatic, and order him to be conveyed to the lunatic asylum for restraint and treatment.

THE EXECUTION OF JUDGMENT.

The law further provides that immediately after any person is adjudged a lunatic the county judge shall communicate with the superintendent of the insane asylum, and if notified by the latter that there is a vacancy in the asylum, or that the patient can be accommodated he shall issue his warrant to the sheriff or some other suitable person directing that the insane person be conveyed to the asylum. The law further provides that before actually conveying the accused to the asylum that the patient be provided with two full suits of substantial summer clothing and one suit of substantial winter clothing.

Now, the above provisions, except of course the provision creating the lunatic asylum and providing for its maintenance, etc., cover the law in Texas governing the charge or information, the trial, the conviction and the judgment and execution of such judgment of one found to be a lunatic.

DEFECTS.

Portions of this law have been on the statute books since 1858, and like all other laws, to the casual reader, seem fair, equitable and just, both to the accused, to those interested in the accused and to the State. In fact, the law makes pretty good reading, and to one reading it and one considering it for the first time without having actually come into contact with its provisions in action, would be overwhelmed with pride because of its munificent provisions and its broad spirit toward our unfortunate insane. But when we come closer to the law and see the actual workings of it, we find it defective from beginning to end and so uncertain in many of its provisions as to leave its enforcement or nonenforcement almost exclusively to the whim or fancy of this jury or that judge without regard to a fixed policy on the part of the State government.

REMEDIES.

In the first place, instead of waiting for information or complaint, would it not be better for the county judge to take judicial notice of the lunatics of the county or pass the matter up to the grand jury and have them investigate each and every cause of complaint?

In the second place, when the matter is brought before the court by any satisfactory method, instead of leaving the case to be judged by a jury or six "competent men," as provided by the statutes, leaving the sheriff to decide who "competent men" are, would it not be far better to have a standing commission composed of say four or five doctors of the county who are competent in the real sense of the word, and let them act as an advisory board with the county judge in all such cases?

What does the ordinary juror know about the technical phases of insanity?

The only way he has of determining whether a man is insane or not is by his appearance and by the testimony of witnesses, while a doctor would be equally competent to consider these phases of the testimony and in addition to that have the benefit of his technical knowledge to aid him in forming a just judgment. In other words, some men who act crazy might be sound of mind so far as practical citizenship is concerned, and the others whose actions are not suspicious in the least might be very dangerous characters in the community. So it occurs to me that this doctors' commission in every county could occupy practically the same attitude towards the county judge as the board of pardons does to the Governor and sit with him in all cases coming up for trial. This method, though crudely outlined, could be worked out so the accused and the State could have a much more satisfactory way of arranging a just judgment in each case.

In the third place, after convictions by the jury under our present law, many abuses have crept in. The law reads that the patient shall be ordered conveyed to the lunatic asylum for restraint and treatment, and further on requires the county judge to communicate with the superintendent of the asylum to see whether or not there is a vacancy. This looks all right in print, but it is the great shame of Texas that under these provisions of the law, plain as they seem, the county jails of almost every county have one or more unfortunate insane patients confined as common lunatics awaiting a vacancy in the asylums. Sometimes it requires months, and I understand a year to get these patients placed. In the meantime they are languishing in the county jails among criminals and surrounded by every unfavorable condition imaginable, many of them dying from diseases contracted and aggravated by the conditions of imprisonment. Those who have insane in their families have a horror of just such results, and many who should be confined and who could be benefited by confinement and possibly cured are left at large because the ones interested in their welfare will not stand for such treatment.

The person of unsound mind or *non compos mentis* is of no further benefit to society or mankind. The asylums are to restrain and cure. If the patient can not be cured, he should be restrained and, if the institutions we now have to accommodate the unfortunate of our State do not accommodate them, it seems that the Legislature should be prevailed upon to provide sufficient accommodations, that all may be taken care of. These people should become wards of the State and should all be placed under State care and treatment, and, in my opinion, a large part of the responsibility for the lax dealings with patients now prevailing in the State is due to the careless neglect on the part of the practicing physicians who are brought into daily touch with the present conditions and evils springing therefrom. They should agitate the matter until the public conscience is awakened.

REFORMS.

1. Have the information more liberal.
2. Provide for a proper trial by a commission of physicians.
3. Prevent confinement in the county jail.
4. Provide for immediate conveyance to the insane asylum.
5. By all means urge the Legislature to provide the proper accommodations.

